# Epigenetic Properties of Compounds Contained in Functional Foods Against Cancer

**DOI:** 10.3390/biom15010015

**Published:** 2024-12-26

**Authors:** Giulia Casari, Brenda Romaldi, Andrea Scirè, Cristina Minnelli, Daniela Marzioni, Gianna Ferretti, Tatiana Armeni

**Affiliations:** 1 Department of Clinical and Specialist Sciences (DISCO), Università Politecnica delle Marche, 60131 Ancona, Italy; g.casari@staff.univpm.it (G.C.); b.romaldi@pm.univpm.it (B.R.); g.ferretti@staff.univpm.it (G.F.); 2 Department of Life and Environmental Sciences (DISVA), Università Politecnica delle Marche, 60131 Ancona, Italy; a.a.scire@staff.univpm.it (A.S.); c.minnelli@staff.univpm.it (C.M.); 3 Department of Experimental and Clinical Medicine, Università Politecnica delle Marche, 60131 Ancona, Italy; d.marzioni@staff.univpm.it

**Keywords:** epigenetic, cancer, phytochemicals, flavonoids, DNA-methylation, histone-modifications, non-coding RNAs

## Abstract

Epigenetics encompasses reversible and heritable genomic changes in histones, DNA expression, and non-coding RNAs that occur without modifying the nucleotide DNA sequence. These changes play a critical role in modulating cell function in both healthy and pathological conditions. Dysregulated epigenetic mechanisms are implicated in various diseases, including cardiovascular disorders, neurodegenerative diseases, obesity, and mainly cancer. Therefore, to develop innovative therapeutic strategies, research for compounds able to modulate the complex epigenetic landscape of cancer is rapidly surging. Dietary phytochemicals, mostly flavonoids but also tetraterpenoids, organosulfur compounds, and isothiocyanates, represent biologically active molecules found in vegetables, fruits, medicinal plants, and beverages. These natural organic compounds exhibit epigenetic modulatory properties by influencing the activity of epigenetics key enzymes, such as DNA methyltransferases, histone acetyltransferases and deacetylases, and histone methyltransferases and demethylases. Due to the reversibility of the modifications that they induce, their minimal adverse effects, and their potent epigenetic regulatory activity, dietary phytochemicals hold significant promise as antitumor agents and warrant further investigation. This review aims to consolidate current data on the diverse epigenetic effects of the six major flavonoid subclasses, as well as other natural compounds, in the context of cancer. The goal is to identify new therapeutic epigenetic targets for drug development, whether as stand-alone treatments or in combination with conventional antitumor approaches.

## 1. Introduction

Epigenetics investigates reversible yet heritable genomic modifications at both the DNA and histone protein levels, regulating gene expression and phenotypic outcomes without modifying the nucleotide sequence itself. These epigenetic changes are intricately linked to chromatin condensation states, which are regulated by histone post-translational modifications and gene methylation patterns that control gene silencing [1]. Epigenetics plays a crucial role in modulating cellular functions in both healthy conditions (including gene expression of each tissue, X-chromosome inactivation, and non-coding DNA modulation) and pathological conditions (such as cancer, Alzheimer’s disease, and neurological diseases) [2]. In both health and disease, gene expression regulation is mediated mainly by DNA methylation processes, covalent histone modifications, and noncoding RNA (ncRNA) activity, all of which have a marked impact on cellular homeostasis [3].

Cancer, as well as other pathologies, represents a complex condition whose etiology, development, and progression are strictly related to both genetic alterations and microenvironment-connected epigenetic factors. Epigenetic mechanisms can directly modulate the expression of proto-oncogenes and tumor suppressor genes, frequently occurring in the early steps of tumorigenesis, often preceding genetic mutations. These features make epigenetic targets highly promising for cancer prevention and therapy [4].

Environmental stimuli, including the intake of dietary phytochemicals, can exert a strong influence on regulating these epigenetic processes [2]. Dietaries are bioactive plant-derived molecules that exhibit several biological effects, such as anticancer, antioxidant, and immunomodulatory features, also thanks to their epigenetic modulation mechanisms. These compounds exert their effects via complementary and overlapping mechanisms, such as the activation of detoxification enzymes and inhibition of nitrosamine formation. Specifically, they target the activity of key epigenetic regulators, including DNA methyltransferases (DNMTs) and histone deacetylases (HDACs) [5,6].

Flavonoids, the most abundant class of natural polyphenolic compounds consumed daily, are particularly effective at modulating several epigenetic processes implicated in cancer and other diseases. Flavonoids are categorized into six subclasses: flavan-3-ols, flavonols, flavones, flavanones, isoflavones, and anthocyanidins, whose roles as epigenetic modulators are extensively reviewed here [7]. Together with flavonoids, other dietary phytochemicals present in vegetables, fruits, beverages, and spices also act as potent epigenetic modulators by inhibiting DNMTs, modifying histones, and modulating micro-RNA (miRNA) expression. Examples include curcumin, lycopene, organosulfur compounds, phenethyl isothiocyanate, and resveratrol, which are highlighted in this review.

Given their properties, these dietary compounds hold potential as epigenetic modulators for use in cancer prevention and therapy, either as standalone treatments or in combination with conventional pharmacological approaches [8]. Starting from these data from the literature, recent research progress concerning epigenetic properties of compounds contained in functional foods, including all types of flavonoids but also other phytochemicals like resveratrol, lycopene, and curcumin, is reviewed here. The focus is on their potential for tumor treatment and prevention, aiming to promote the development of innovative strategies and mitigate the numerous adverse effects associated with traditional approaches.

## 2. Epigenetic Mechanisms

**Figure 1:**
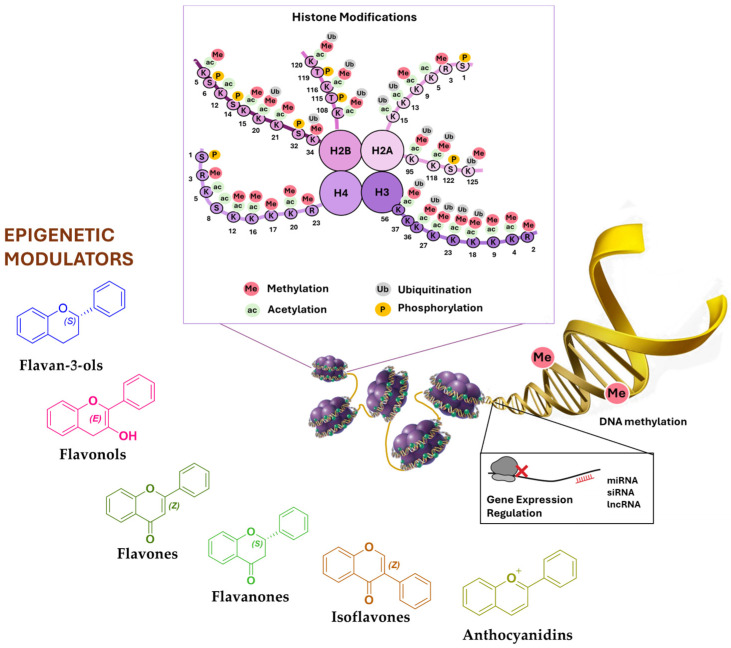
Summary of key epigenetic mechanisms affecting the development and progression of tumors and the chemical scaffold of the main flavonoids acting as epigenetic modulators. Epigenetic mechanisms, including DNA methylation and histone modifications like methylation, acetylation, phosphorylation, and ubiquitination, regulate the transcriptional activity of genes. Natural epigenetic modulators, such as flavan-3-ols, flavonols, flavones, flavanones, isoflavones, and anthocyanidins, which influence these epigenetic modifications, are highlighted. The image also includes epigenetic modifications mediated by ncRNAs, such as miRNAs, siRNAs, cirRNA, and lncRNAs, suggesting that phytochemicals may exert a modulating role through these epigenetic mechanisms.

Among the several epigenetic mechanisms, DNA methylation, post-translational histone modifications (e.g., phosphorylation, deacetylation, methylation), and expression variation in ncRNAs (e.g., siRNA, miRNA, lncRNA, cirRNA) represent the most epigenetic modulators on which phytochemicals could exert their potential [9] (Figure 1). These reversible epigenetic alterations play a critical role in the onset, development, and progression of tumors. Indeed, abnormal promoter hypermethylation, aberrant histone acetylation, and miRNA expression dysregulation can promote improper gene silencing within each step of tumor progression [10].

### 2.1. DNA Methylation

In DNA methylation, a methyl group (-CH_3_) is transferred 5′ to a cytosine base, particularly when it precedes a guanine in a CpG dinucleotide, to form 5-methyl cytosine. This occurs most frequently in CpG islands, regions of DNA with a high frequency of CpG sites, which are often located in gene promoter regions and enhancers. CpG islands are typically characterized by a cytosine and guanine content greater than 50% and a sequence length greater than 200 base pairs. Methylation of these regions often leads to gene silencing, playing a vital role in regulating gene expression during development and in disease processes such as cancer [11]. Although CpG methylation is the most well-studied in mammals, DNA methylation can also occur at other bases, such as adenine, to form N6-methyladenine. DNA methylation is mediated by a group of enzymes called DNA methyltransferases (DNMTs), which includes the isoenzymes DNMT1, DNMT3a, and DNMT3b [12]. DNMT1 represents the main enzyme responsible for the preservation of DNA methylation sites through the maintenance of existing DNA methylation patterns during DNA replication. This enzyme is essential for the inheritance of epigenetic marks, ensuring that methylation patterns are copied to daughter cells after cell division. DNMT3a and DNMT3b, deeply expressed in embryogenesis and slightly expressed in adults, are mainly involved in de novo methylation as they can add methyl groups to previously unmethylated DNA sequences. These enzymes play a key role in early embryogenesis, where widespread DNA methylation is essential for normal development [13]. Moreover, there is also DNMT-3L, which facilitates retrotransposon methylation through the interaction between DNMT3a and DNMT3b [14]. Aberrant DNA methylation patterns are a hallmark of cancer. Indeed, in tumor cells, the CpG methylation pattern significantly differs from that of healthy cells and is often deregulated. The promoters of oncogene are often characterized by hypomethylation in CpG islands, resulting in their overexpression and uncontrolled cell proliferation. Instead, promoters of tumor suppressor genes frequently are hypermethylated, leading to gene silencing. This prevents these genes from performing their normal role in halting tumor cell growth, thus promoting the survival and expansion of cancerous cells [15]. Moreover, aberrant DNA methylation affects genes implicated in apoptosis, migration, and DNA repair, all of which contribute to tumor progression and metastasis [16,17]. The reversible nature of DNA methylation makes it an attractive target for cancer therapy. Therefore, compounds able to inhibit DNMTs can induce the re-expression of silenced genes, such as tumor suppressor genes, thus playing an anticancer role. Indeed, several DNMT inhibitors (DNMTi) are currently being investigated within clinical trials or are already used in clinical practice against cancer, given their ability to reverse aberrant methylation patterns. By inhibiting DNMT activity, these compounds can reactivate silenced tumor suppressor genes, restoring their function and thereby inhibiting tumor growth.

However, DNMT inhibitors are often associated with toxicity, which creates a major limitation to their use in clinical settings. The Food and Drug Administration (FDA) has approved two DNMT inhibitors, Azacitidine (Vidaza) and Decitabine (Dacogen) [18], for the treatment of certain types of cancer, particularly myelodysplastic syndromes and acute myeloid leukemia. Both drugs incorporate into DNA and inhibit DNMT activity, leading to the passive demethylation of DNA during cell division. However, their effectiveness can be compromised by side effects, such as myelosuppression, resulting in neutropenia, thrombocytopenia, and other toxicities. Among the most frequent nonhematological effects are gastrointestinal toxicities, including diarrhea, vomiting, nausea, and constipation, which are usually moderate and transient and easily treated with antiemetics and antidiarrheal drugs [18,19]. In addition to synthetic DNMT inhibitors, several natural compounds, particularly flavonoids, and other plant-derived molecules, have been shown to be promising as DNMT inhibitors. These compounds may modulate DNMT activity either directly or indirectly, offering a potentially less toxic alternative to synthetic inhibitors. Further research is needed to evaluate their therapeutic potential and safety in humans [20,21].

### 2.2. Histone Modifications

Histones are positively charged proteins involved in DNA condensation processes into chromatin within the nucleus. Histones have tails, particularly at their N-terminus, that are subject to various post-translational modifications (PTMs) (Figure 1). These PTMs include both de/acetylation and de/methylation of the histone N-terminal, as well as phosphorylation. They directly influence chromatin structure and, thus, the accessibility of DNA to transcription factors, thereby affecting gene expression [7,22]. Histone acetylation and deacetylation are among the most studied histone modifications, playing a pivotal role in gene expression regulation.

Histone deacetylation is regulated by HDACs, divided into zinc/iron-dependent HDACs and NAD^+^-dependent ones [23]. Histone acetylation, mediated by histone acetyltransferases (HATs), consists of adding an acetyl group to lysine residues at the histone N-terminal. This PTM neutralizes the positive charge of lysine, reducing the affinity between histones and the negatively charged DNA. This results in a relaxed chromatin structure and allows for transcriptional activity [24]. Instead, the histone deacetylation of *e*-amino groups of lysine residues, mediated by HDACs, inhibits gene expression by compacting chromatin structure [25]. HDACs are classified into two main categories: Zinc/iron-dependent HDACs, which require metal ions for their activity, and NAD+-dependent HDACs, which utilize the NAD+ molecule for their enzymatic function [23].

The pattern of acetylation and deacetylation is markedly different between healthy and tumor cells [26]. Tumor cells frequently exhibit aberrant deacetylation of histones, resulting in the silencing of tumor suppressor genes and promoting malignant growth. Consequently, HDAC inhibitors (HDACi) have emerged as promising agents in cancer therapy because they can restore the expression of these silenced genes. Several HDACi have shown antitumor activity in both preclinical studies and clinical trials (reviewed in [27]), including Tucidinostat for breast cancer and Entinostat for advanced epithelial ovarian cancers. Although distinct HDACi have shown promising results, they display low specificity to the different HDAC isoforms, thus leading to several adverse effects such as cardiotoxicities, as well as hematologic and gastrointestinal alterations. This highlights the need to improve the therapeutic efficacy of these drugs through the development of more selective HDACi. In this regard, natural compounds like flavonoids have been investigated for their HDAC inhibitory activity, offering a potential alternative with fewer side effects [20,28].

Histone de/methylation mostly occurs on arginine and lysine residues within the tails of histone proteins by histone methyltransferases (HMTs) and histone demethylases (HDMs) and plays a key role in modulating transcriptional activation or gene silencing in cancer [29]. HMTs add methyl groups to histones, influencing gene expression. Unlike acetylation, methylation does not affect the charge of histones but instead creates a binding platform for other regulatory proteins. HDMs remove methyl groups, reversing the effects of methylation and dynamically regulating gene expression. The functional outcome of histone methylation depends on the methylated residue and its degree of methylation (mono-, di-, or tri-methylation). For instance, H3K4 methylation is associated with active transcription, while H3K9 and H3K27 methylation are linked to gene repression and heterochromatin formation [29]. Dysregulation of histone methylation is common in cancer. For example, overexpression of EZH2, a methyltransferase that methylates H3K27, leads to the silencing of tumor suppressor genes and is implicated in the progression of several cancers. Targeting the enzymes that mediate histone methylation and demethylation has become an area of intense research in cancer therapy.

Finally, histone phosphorylation represents another epigenetic modification carried out by cell-cycle-related kinases. Histone phosphorylation is typically associated with chromatin remodeling during processes such as DNA damage repair, cell division, and apoptosis. This modification is mediated by various kinases and occurs primarily on serine, threonine, and tyrosine residues of histones. Phosphorylation can alter the interaction between histones and DNA, facilitating access to repair proteins and other factors [30]. During mitosis, phosphorylation of H3S10 is crucial for chromatin condensation.

### 2.3. Non-Coding RNAs

An important regulation of gene expression at the post-transcriptional level is mediated by non-coding RNAs (ncRNAs). NcRNAs are a group of transcripts that are not translated into proteins but exert specific functions at the RNA level. Among the regulatory ncRNAs, there are miRNAs, small interfering RNAs (siRNAs), long non-coding RNAs (lncRNAs), and circular RNA (cirRNA) with crucial biological functions. MiRNAs are small ncRNAs of 18–25 nucleotides, able to influence both mRNA stability and translation-inducing RNA-silencing through the interaction with the mRNA 3′ region. Different genes can be modulated by the same miRNAs, and several miRNAs can regulate the transcription profiles of the same gene, creating a complex regulatory network that fine-tunes gene expression [31,32]. MiRNAs play a crucial role in various physiological processes, including cell differentiation, proliferation, apoptosis, and development. Similarly, cirRNAs have emerged as important regulators of gene expression. By sequestering miRNAs, cirRNAs prevent them from binding their mRNA targets, influencing mRNA stability and translation [33]. However, their dysregulation is a characteristic feature of several diseases, including cancer. Different miRNAs, called onco-miRNAs, play a crucial role in cancer growth and progression by influencing the transcription of both oncogenes and tumor suppressor genes because their misexpression has been related to tumorigenesis processes [34]. A notable example is miR-21, one of the most studied onco-miRNAs, which is frequently overexpressed in various cancers, including breast, lung, and colon cancers. MiR-21 promotes tumor growth by targeting tumor suppressors such as *PTEN* and *PDCD4* [35].

In addition to miRNAs, lncRNAs are polyadenylated RNAs that last longer than 200 nucleotides and are able to interact with DNA, RNA, and proteins. LncRNAs participate in gene regulation at multiple levels, including chromatin remodeling, transcriptional activation/repression, splicing regulation, and mRNA stability [36]. LncRNAs are known to function as scaffolds, decoys, or guides in the regulation of gene expression. Scaffold lncRNAs serve as platforms for the assembly of protein complexes, facilitating interactions between proteins or between proteins and chromatin. Decoy lncRNAs bind and sequester transcription factors or other regulatory proteins, preventing them from interacting with their target genes. Guide lncRNAs direct chromatin-modifying complexes to specific genomic loci to influence transcription [37].

In the context of cancer, lncRNAs can act as tumor suppressors or oncogenes by regulating a wide range of pathways underlying cancer [38]. For example, HOTAIR is a well-characterized oncogenic lncRNA that is overexpressed in various cancers, including breast cancer, where it promotes metastasis by altering the epigenetic landscape through interaction with PRC2 (Polycomb Repressive Complex 2) [39]. Conversely, lncRNAs like MEG3 function as tumor suppressors by enhancing p53 activity and promoting cell cycle arrest [40].

The misregulation of ncRNAs is a common feature of cancer. CirRNAs are also strongly implicated in tumor biology. Dysregulated expression of cirRNAs may contribute to oncogenesis by acting as competitive endogenous RNAs (ceRNAs) for onco-miRNAs or by modulating signaling pathways for tumor growth and metastasis [33]. Recent studies have shown that cirRNAs play a role in the regulation of epigenetic enzymes such as HDACs and DNMTs. Behaving as competing endogenous RNAs (ceRNAs), cirRNAs bind and sequester miRNAs, thus indirectly influencing the expression and activity of these epigenetic enzymes. This regulatory mechanism is crucial in diseases such as cancer and neurodegenerative disorders, where changes in HDAC and DNMT activity lead to altered gene expression and contribute to disease progression [41]. CirRNAs can influence signaling pathways involved in cell proliferation, apoptosis, and metastasis. In particular, they regulate the expression of oncogenes and tumor suppressors through interaction with specific miRNAs, and their alteration may contribute to gene dysregulation and tumorigenesis [42]. Both miRNAs and lncRNAs are involved in the initiation, progression, and metastasis of tumors by modulating the expression of oncogenes and tumor suppressor genes [34,43]. Anyway, ncRNAs are promising targets for therapeutic intervention. Therapies targeting cirRNAs are also being explored, focusing on either restoring normal cirRNA expression or inhibiting oncogenic cirRNAs that contribute to tumor progression [44]. MiRNA mimic drugs and anti-miRNAs (antagomirs) are being developed to restore normal miRNA function in cancer cells [45]. Similarly, lncRNA-based therapies are being investigated, including approaches to silence oncogenic lncRNAs or restore the function of tumor-suppressive lncRNAs [46].

## 3. Phytochemicals Targeting Enzymes Involved in Epigenetic Regulation

**Figure 2:**
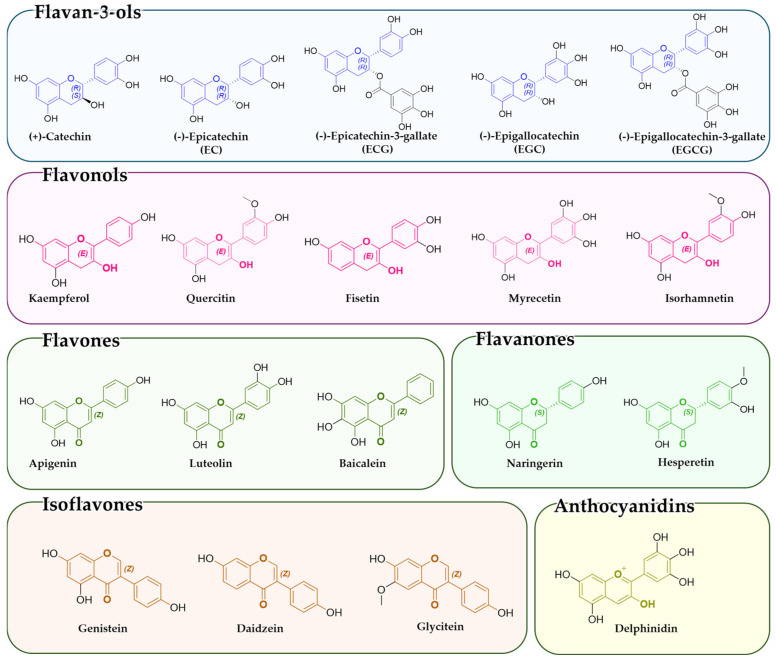
2D representations of various classes of flavonoids, with the characteristic central core highlighted in different colors.

Among phytochemicals, flavonoids are natural polyphenolic compounds characterized by the flavan backbone, which consists of two phenolic rings linked by an oxygen-containing heterocycle (Figure 2). These plant-derived metabolites, although not produced within the human body, can be consumed daily by eating different fruits, vegetables, nuts, cereal, and beverages like coffee and tea [47,48]. Flavonoids are responsible for several health benefits for mammals in the nutraceutical, medical, and pharmaceutical fields. Indeed, they have pro-apoptotic, antibacterial, antiviral, and analgesic properties through a wide range of molecular processes, like epigenetic mechanisms, cell cycle modulation, and regulation of detoxification enzyme activities [48,49]. Furthermore, flavonoids show anticancer potential by both inhibiting oncogene expression and promoting the expression of tumor suppressor genes [50,51]. This modulation also occurs at the epigenetic level, inhibiting the activities of several enzymes involved in epigenetic regulation, like HATs, HDACs, and DNMTs, and modulating the expression of ncRNAs [4]. A study also explored the correlation between cirRNAs, polyphenols, and cancer, showing that polyphenols, known for their anti-cancer properties, can influence cirRNA expression. Polyphenols such as resveratrol and curcumin have been observed to modulate certain cirRNAs, with significant effects on cancer cell behavior, including the regulation of pathways associated with cell cycle control and apoptosis. This interaction is particularly relevant for cancer therapies, where modulation of cirRNAs could improve treatment efficacy or overcome drug resistance [52,53].

Flavonoids, thanks to their lower toxicity against healthy and their few side effects compared to the traditional treatments, alone or combined with other drugs, might be an attractive strategy to treat cancer conditions, even if additional studies of safety and efficacy are required [7,20,54]. The potential of flavonoids as anti-tumor agents has been extensively studied, as demonstrated by the increasing number of publications related to the synthesis of flavonoid derivatives with improved biological activities. These modifications are aimed at enhancing their interaction with specific tumor targets such as EGFR [55] or increasing their selective toxicity towards tumor cells as opposed to healthy ones [56]. Additionally, researchers have focused on optimizing the structure of flavonoid compounds to improve their ability to selectively inhibit the activity of enzymes involved in epigenetic regulation, such as HDAC [57] and DNMT inhibitors [58].

According to their chemical structures, flavonoids can be divided into the following six groups: flavan-3-ols, flavonols, flavones, flavanones, isoflavones, and anthocyanidins (Figure 2) [59].

Flavan-3-ols (also known as catechins) are characterized by the absence of a double bond between the C2 and C3 carbons, which differentiates them from other flavonoids. Key compounds in this subclass, such as catechins and epigallocatechin gallate (EGCG), are commonly found in green tea and cocoa. Flavan-3-ols have several properties, including anticancer potential, particularly EGCG from green tea. EGCG induces apoptosis, inhibits tumor proliferation, reduces inflammation, and epigenetically modulates DNMTs, HATs, and miRNA, reactivating tumor suppressor genes [60,61,62]. Other flavan-3-ols, such as EGC and ECG, also show similar effects, making them promising cancer therapeutic agents [20,63,64].

Another important subclass is the flavonols, which are structurally similar to flavones but differ by having a hydroxyl group attached to the C3 position. Quercetin and kaempferol, for example, are found in foods like onions, apples, and green tea. These compounds exhibit antioxidant, anti-inflammatory, and anticancer properties, acting through epigenetic regulation, including inhibition of DNMTs, HDACs, and HMTs and modulation of tumor suppressor genes and miRNAs [65,66].

The flavones subclass, characterized by a double bond between the C2 and C3 positions in the central ring, have anticancer pharmacological properties due to their ability to epigenetically regulate gene expression. Apigenin, for example, inhibits the activity of HDACs and DNMTs and promotes cell cycle arrest and apoptosis in cancer cells [67,68]. Luteolin has a similar role by modulating miRNA expression and inhibiting metastasis in breast cancer [4,69].

Flavanones subclass, which differs from flavones by having a saturated bond between C2 and C3, are particularly stable and primarily found in citrus fruits. Compounds such as naringenin and hesperetin inhibit the activity of HDACs and DNMTs, promoting apoptosis and antioxidant activity [70]. Also, hesperidin exhibits neuroprotective, anti-inflammatory, and cardioprotective effects, making them essential for overall health.

Isoflavones are noteworthy due to their structural similarity to human estrogens. Isoflavones, found in legumes such as soya, have phytoestrogenic and anticancer properties. They work by inhibiting DNMT enzyme activity, activating HATs, and modifying DNA methylation, with beneficial effects against prostate and breast cancer. Genistein and daidzein act as phytoestrogens, influencing hormonal health, preventing osteoporosis, and reducing the risk of certain chronic diseases. Genistein, in particular, also regulates miRNA expression and increases estrogen receptor α expression [71,72,73,74].

Anthocyanins are responsible for the red, purple, and blue colors in many fruits and vegetables. Compounds like cyanidin and malvidin, found in berries, grapes, and eggplants, are recognized for their antioxidant and anti-inflammatory properties. Anthocyanins act by activating the Nrf2-ARE pathway and promote apoptosis in cancer cells by regulating methylation and HDAC activity [75,76].

### 3.1. Flavan-3-ols

Flavan-3-ols are a subclass of flavonoids with two chiral carbons in the central ring. They include catechin and epicatechin (EC), which are the simplest monomers, and epicatechin-3-gallate (ECG), epigallocatechin (EGC), and epigallocatechin-3-gallate (EGCG), which are formed by esterification at position 3 of the central ring. High levels of flavan-3-ols are present in tea, wine, apples, grapes, and cacao. In particular, EC, ECG, EGC, and EGCG are the major polyphenols in green tea, whereas catechins and thearubigens are the major ones in black tea [77].

Among these flavan-3-ols, EGCG stands out, representing more than 50% of total flavan-3-ols in green tea. EGCG promotes apoptosis in cancer cells through various mechanisms, including the inhibition of the Epidermal Growth Factor receptor (EGFR) [78], the activation of caspase (specifically, caspase 9, 8, and 3), and the upregulation of pro-apoptotic proteins such as Bax. Furthermore, it induces cell cycle arrest by enhancing the expression of cell cycle regulator proteins, like p21 and p27, thereby inhibiting tumor cell proliferation while preserving healthy cells. It also reduces oxidative stress, angiogenesis, and inflammation and exhibits antiviral properties (Table 1) [20]. These multifaceted actions help to prevent the formation of new blood vessels, which are crucial for tumor growth.

At the epigenetic level, it has been demonstrated that EGCG inhibits the activity of DNMTs (particularly DNMT1) in several tumor cell lines, including breast, colon, prostate, and esophageal tumor cells, thereby promoting the reactivation of antioxidant enzymes. Breast cancer cells (but also lung, oral cavity, liver, and thyroid tumor cells) treated with EGCG showed a reduced level of telomerase, a key enzyme involved in cellular immortality, due to the decreased methylation of *human telomerase reverse transcriptase (hTERT)* promoter, thus leading to a cell growth inhibition and an enhancement in the sensitivity to chemotherapy [61]. In both estrogen receptor (ER)-positive (MCF-7) and ER-negative (MDA-MB-231) breast cancer cells, EGCG also increases the binding of hTERT repressor E2F-1 to its promoter [79]. Moreover, by inhibiting DNMT activity, EGCG demethylates *signal peptide CUB-EGF-domain-containing protein 2 (SCUBE2)* promoter, revoking the epithelial-mesenchymal transition and thus easing tumor progression [80]. Along with the modulation of DNMT activity, EGCG also regulates HAT activity, thus playing a crucial role in chromatin reorganization. This regulation can lead to the re-expression of various tumor suppressor genes that are often silenced in cancer cells [60]. “In vivo” studies also demonstrated that EGCG could modulate miRNAs in cancers such as hepatocellular carcinoma and gastric cancer, targeting critical genes like *c-Kit*, *Bcl2*, *E2F*, and *RAS*. The dysregulation of these genes is frequently associated with cancer progression, and EGCG’s ability to modulate their expression through miRNAs underscores its potential as a therapeutic agent [62]. Regulation of DNMTs, HATs, and miRNAs alters the expression of different tumor suppressor genes. Taken together, data obtained from both in vitro and in vivo studies suggest that EGCG has proapoptotic, antiangiogenic, and, therefore, anti-cancer potential through its action as a broad epigenetic modulator.

Other flavan-3-ols found in green and black tea, including gallocatechin, EC, ECG, EGC, and thearubigens, showed similar effects compared to EGCG in promoting tumor cell apoptosis, inhibiting growth and reactivating tumor suppressor genes in different cancer cell lines (Table 1) [7]. For instance, EGC showed the ability to inhibit prostate cancer cell growth and induce apoptosis by regulating several signaling pathways [7,20].

**Table 1:** Epigenetic effects of flavan-3-ols and their role in pathological conditions.

Compounds FLAVAN-3-OLS	Epigenetic Effects	High Content Food Quantity mg/100 g [81]	Role in Pathological Conditions
(−)-Epigallocatechin 3-gallate (EGCG) SubClass: Flavan-3-ols Class: Flavonoids Family: Polyphenols	Inhibition of DNMT activity (mainly DNMT1) in breast, colon, prostate, and esophageal cancer cells [61] Inhibition of *hTERT* promoter methylation in breast, lung, oral cavity, liver, and thyroid tumor cells [61] Demethylation of *SCUBE2* promoter, inhibiting the epithelial-mesenchymal transition [80] Modulation of HAT activity [60] Regulation of miRNA expression in hepatocellular carcinoma and gastric cancer in vivo, targeting *c-Kit*, *Bcl2*, *E2F*, and *RAS* [62]	Carob Flour (109.40), Green tea (70.20), White tea (42.45), Oolong tea (34.48), Black tea (9.36), Nuts pecans (2.30), Fuji Apple with skin (1.93), Hazelnut (1.06), Cranberry (0.97), Blackberry (0.68), Raspberry (0.54), Plum (0.48), Pistachio nuts (0.40), Peach (*Prunus persica*) (0.30), Granny Smith Apple, with skin (0.24), Golden Delicious Apple, with skin (0.19), Pear (0.19), Avocado (0.15), Red Delicious Apple, with skin (0.13), Gala Apple, with skin (0.11), Strawberry (0.11)	Reduction in oxidative stress, angiogenesis, and inflammation in tumors [82] Cancer prevention [83,84] Induction of apoptosis by activating caspase 9, 8, and 3 and upregulating Bax protein in cancer cells [85] Induction of cell cycle arrest by enhancing p21 and p27 expression in tumor cells [85]
(−)-Epicatechin (EC), Epicatechin-3-gallate (ECG) Epigallocatechin (EGC) SubClass: Flavan-3-ols Class: Flavonoids Family: Polyphenols	Inhibition of DNMT activity [64] Regulation of HAT activity [20] Modulation of miRNA expression in cancer [63]	**(−)-Epicatechin:** Cocoa powder (158.3) Baking chocolate (141.83), Cacao seed (99.18), Grape seed (93.31), Dark chocolate (84.80), Soybean, mature seed (37.41), Broad beans, immature seed (*Vicia faba*) (28.96), Apple, skin only (28.73), Blueberry (25.66), Green tea, Quingmao (20.80), Sour cherry juice (12.97), Milk chocolate (10.88), Alcoholic beverage, red wine (10.66–3.79), Apple (9.83–4.09), Grape, black (*Vitis vinifera*) (8.68), Cherry (*Prunus avium*) (5.00), Apricot (*Prunus armeniaca*) (4.74), Apple juice (4.70), Blackberry (4.66), Cranberry (4.37), Peache, white, (4.09), Pear, raw (*Pyrus communis*) (3.76), Raspberry (*Rubus* spp.) (3.52), Plum (3.20), Nectarine (*Prunus persica*) (3.06–2.34), Buckwheat flour, whole-groat (3.02), Cranberry bush berries (*Viburnum opulus*) (2.69), Oolong tea (2.54), Vinegar, wine, red (2.20), Black tea (2.13), Arctic bramble berries (1.80), Grape, white (1.70), Strawberry tree fruit (*Arbutus unedo*) (1.56), Pistachio nuts (*Pistacia vera*) (0.83), Nut pecans (*Carya illinoinensis*) (0.82), Vinegar, cider (0.82), Cloudberry (0.80), Kiwifruit, gold (*Actinidia chinensis*) (0.64), Nut almonds (*Prunus dulcis*) (0.60), Alcoholic beverage, white wine (0.55), Medlar (*Mespilus germanica*) (0.53), Rhubarb (*Rheum rhabarbarum*) (0.51), Fig (*Ficus carica*) (0.50), Currants, European black (*Ribes nigrum*) (0.47) **Epicatechin-3-gallate and Epigallocatechin:** Cocoa powder (196.43), Cacao Bean (99.18), Carob Flour (30.06), Green tea (8.33), White tea (8.35), Oolong tea (2.54), Black tea (2.11), Nut almond (0.60), Nut pecan (0.82)	Antioxidative, anti-inflammatory, and anticancer effects [20]
(+)-Catechin, (+)-Gallocatechin SubClass: Flavan-3-ols Class: Flavonoids Family: Polyphenols	Inhibition of DNMT activity [64] Regulation of HAT activity [20] Modulation of miRNA expression in cancer [63]	**(+)-Catechin:** Blueberry (98.47), Cacao Bean (88.45), Grape seed (74.63), Tea green, Quingmao (67.60), Cacao powder (64.82), Carob Fluor (50.75), Blackberry (37.06), Cranberry (29.04), Chocolate, dark (24.20), Cocoa powder (21.51), Plum Black Diamond(17.22), Broad bean (*Vicia faba*) (14.29), Peache white (12.25), Grape Black (10.14), Nectarine (*Prunus persica*) (7.58), Apple skin only (7.40), Nut Pecan (7.24), Alcoholic beverage, wine red (from 6.21 to 7.70) Strawberry (6.70), Chard, red leaf (*Beta vulgaris*) (6.70), Apple (6.67–0.75) Banana (6.10), Blueberry (5.29), Bean, pinto (*Phaseolus vulgaris*) (5.07). Cider (4.85), Green tea (4.47), Cherry (4.36), Milk chocolate (4.16), Grape, white (3.73), Apricot (3.67), Vinegar, wine (3.60), Pistachio nuts, (3.57), Jujube (*Ziziphus jujuba*) (3.21), Strawberry (3.11), Juice sour cherry (3.18), Plum (2.89), Barley, hulled (*Hordeum vulgare* L.) (2.39), Arctic bramble berry (2.30), Rhubarb (*Rheum rhabarbarum*) (2.17), Mango (1.72), Gooseberry (1.67), Raspberry (1.31), Currants, red (1.27), Apple juice (1.25), Black tea (1.51), Nut, hazelnut or peanut (*Corylus* spp.) (1.19), Quinces (0.75) **(+)-Gallocatechin:** Cacao Bean (8262.00), Broad bean, immature seeds (*Vicia faba*) (4.15), Strawberry tree fruit (*Arbutus*) (1.60), Green Tea (1.54), Currants, red (1.28), Black tea (1.25), Pomegranate, raw (0.17), Persimmon (0.17), Star apple (0.53)	Antioxidative, anti-inflammatory, and anticancer effects [20]
Thearubigins SubClass: Flavan-3-ols Class: Flavonoids Family: Polyphenols	Inhibition of DNMT activity [86]	Black tea (81.30)	Antioxidative, anti-inflammatory, and anticancer effects [87,88]

DNMT: DNA methyltransferase; HAT: histone acetyltransferase; miRNA: microRNA.

### 3.2. Flavonols

Flavonols, the most common flavonoids present in food, are characterized by the presence of a 3-hydroxyflavone backbone. The different positions of the phenolic -OH groups give rise to their diversity. Kaempferol, quercetin, fisetin, and myricetin are the main plant-derived flavonols, which can be found in different vegetables and fruits like strawberries, apples, asparagus, and onions but also in wine [89].

Kaempferol, found in *Zingiberaceae* vegetables and fruits, including strawberries, hop, tomatoes, and grapefruit, shows several physiological functions, such as inhibition of fat formation, nervous system, and heart protection, antioxidant, antiallergic, and anticancer effects (Table 2) [87]. Due to its capability to regulate gene expression through epigenetic modification, kaempferol has shown promising results as an anticancer agent. Specifically, it showed the ability to inhibit the activity of different human HDAC enzymes and promote hyperacetylation of histone H3 in hepatoma and colon tumor cell lines [90]. Moreover, in colorectal cancer cell lines, kaempferol can bind DNMT1 and downregulate the methylation of tumor suppressor gene *DACT2*, thus inhibiting the Wnt/β-catenin pathway [90]. In gastric tumor cells, kaempferol promotes autophagic cell death, whereas, in lung tumors, it inhibits cancer cell proliferation and induces cell apoptosis by regulating miR-340 expression [65].

Quercetin, the predominant flavonol present in buckwheat and citrus fruits, has antioxidant, anticancer, anti-inflammatory, antiviral, antibacterial, neuroprotective, and hypolipidemic impacts (Table 2). It reduces the activity of DNMTs, HMTs, and HDACs in cervical tumor cells, along with the global concentrations of DNA methylation, whereas it activates HATs, thus restoring the expression of different tumor suppressor genes [66]. In pancreatic tumor cells, it inhibits cell proliferation by promoting let-7c and, in turn, Numb1 and Notch, and induces apoptosis. It has also been related to anti-inflammatory properties and angiogenesis inhibition [91]. In triple-negative breast cancer, quercetin can control the beta-catenin signal, leading to the reversion of epithelial-mesenchymal transition and the increase in *BRCA1* expression [92]. Combined with curcumin, quercetin further increases *BRCA1* levels by enhancing the histone H3K9 acetylation of the *BRCA1* promoter [93]. Moreover, quercetin regulates different miRNAs, like miR-145 and miR-146a, and regulatory axes including miR-22/WNT1/β-catenin, miR-197/IGFBP5, TP53/miR-15/miR-16, miR-16/HOXA10 and p53/miR-34a/SIRT1 in several tumor cell lines [7]. Inhibition of both proliferation and invasion of breast cancer cells is mediated by quercetin through the upregulation in miR-146a expression and the subsequent activation of caspase-3 and Bax, thus resulting in mitochondrial-dependent apoptotic pathway triggering [94].

Fisetin is a flavonol present in onions, apples, strawberries, tea, and wine. It can interfere with cancer cell growth and cell cycle progression and promote PARP cleavage and apoptosis through DNMT inhibition (Table 2) [95]. Furthermore, fisetin regulates the Bcl-2 family protein expression and inhibits signaling pathways in which p38 MAPK, ERK1/2, or NF-kB are involved [96]. Fisetin, as well as quercetin, activates sirtuins (SIRTs) [97].

Myricetin is contained in berries, grapes, tea, and red wine. It plays a crucial role in the prevention of cardiovascular disorders, has anticancer ability, and reduces blood lipid levels, blood pressure, and diabetes and bacteriostasis complications (Table 2) [4]. It is considered the most potent flavonol, and it inhibits DNMT activity in a concentration-dependent manner [20]. Furthermore, myricetin indirectly modulates deacetylation, activating HDAC SIRT1, such that it can induce *HIF-1α* expression and suppress *cMyc* and *β-catenin* expression [98].

Isorhamnetin is another type of flavonol, specifically an O-methylated flavonol, predominantly found in the fruits and leaves of several plants used in traditional herbal medicine. This compound has garnered attention for its potential benefits in managing diabetes and for its protective effects on the kidneys, which may involve the modulation of epigenetic regulators [99]. However, specific evidence linking isorhamnetin directly to the modulation of epigenetic genes and its direct implications for cancer treatment or prevention may not be well established.

**Table 2:** Epigenetic effects of flavonols and their role in pathological conditions. Content in food [81].

Compounds FLAVONOLS	Epigenetic Effects	High Content Food Quantity mg/100 g	Role in PATHOLOGICAL Conditions
Kaempferol SubClass: Flavonols Class: Flavonoids Family: Polyphenols	Inhibitory activity towards HDAC enzymes in human hepatoma and colon cancer cell lines [65] Promotion of H3 histone hyperacetylation in hepatoma and colon tumor cells [65] Binding to DNMT1 in colorectal cancer cell lines, thus downregulating *DACT2* methylation and inhibiting Wnt/β-catenin pathway [90] Regulation of miR-340 expression in lung tumor [7,66]	Caper (259.19), Caper canned (131.34), Kale (46.80), Arugula (34.89), Mustard green (38.30), Ginger (33.60), Watercress (23.03), Radish (21.85), Chia seed (12.30), Chives (10.0), Chard (9.20), Collards (8.74), Broccoli (7.84), Lovage (*Levisticum officinale*) (7.0), Fennel leaves (6.50), Dried Goji berries (6.20), Cherry powder (5.14), Thistle (3.80), Chicory green (2.45), Corn poppy (*Papaver rhoeas*) (2.30), Blueberry (1.66), Black Tea (1.41), Asparagus (1.39), Bee Pollen granules (1.12), Acerola (1.05), Green Tea (1.00), Ribes Nigrum (0.71), Red onion (0.70), Elderberry (0.51), Strawberry (0.49), Carob Flour (0.44), Grapefruit (0.40), Lingonberry (0.38), Blackberry (0.27), Apple (0.14), Cranberry (0.12)	Inhibition of fat formation, nervous system protection, and heart protection [87] Antioxidant, antiallergic and anticancer effects [87] Promotion of autophagic cell death in gastric tumor cells; inhibition of cancer cell proliferation and induction of cell apoptosis in lung tumor [7,66]
Quercetin SubClass: Flavonols Class: Flavonoids Family: Polyphenols Current dosage: 500 mg quercetin/day, 12 weeks Mean intake by fruits or vegetables: 5–500 mg/day IC_50_: 1.6 µmol/L	Reduction in the activity of DNMTs, HMTs, and HDACs, and activation of HATs in cervical tumor cells [66] Regulation of different miRNAs (like miR-145 and miR-146) and regulatory axes (such as miR-22/WNT1/β-catenin and p53/miR-34a/SIRT1) in several tumor cell lines [7]	Caper (233.84), Caper canned (172.55), Levisticum (170.0), Juice concentrate Elderberry (108.16), Radish (70.37), Arugula (66.19), Corianders leaves (52.90), Peppers yellow wax (50.63), Fennel leaves (48.80), Juniper berries ripe (46.16), Red onion ((39.21), Watercress (29.99), Elderberries (26.77), Corn poppy (*Papaver rhoeas*) (26.30), Carob flour (38.78), Onion cooked boiled drained (24.36), Kale (22.58), Been pollen (20.95), Apple skin (19.36), Chia seed (18.20), Cherries powder (17.44), Thistle (16.50), Arugula (15.16), Asparagu cook boiled-drained (15.16), Cranberries (14.84), Asparagus (13.98), Goji berry dried (13.60), Lingonberries (13.30), Plums black Diamond (12.45), Lovage Mustard green (8.80), Blueberries (7.67), Chard (7.50), Chicory green (6.49), Chives (4.77), Acerola (4.74), Ribes Nigrum (4.45), Apple (4.01), Blackberries (3.58), Broccoli (3.26), Collards (2.57)	Antioxidant, anticancer, anti-inflammatory, antiviral, antibacterial, neuroprotective and hypolipidemic impact [66] Inhibition of cell proliferation by promoting let-7c, Numb1, and Notch, and induction of cell apoptosis in pancreatic tumor cells [91] Regulation of β-catenin signal and promotion of *BRCA1* expression in triple-negative breast cancer [92]
Fisetin SubClass: Flavonols Class: Flavonoids Family: Polyphenols IC_50_: 3.5 µmol/L	Inhibition of DNMT activity [95] Activation of SIRTs [97]	Strawberry (16.0), Apple (2.69), Persimmons (1.05), Onion (0.48), Grapes (0.39), Kiwi (0.2)	Interference with cancer cell growth, cell cycle progression, and promotion of cell apoptosis [95] Regulation of the Bcl-2 family protein expression and inhibition of signaling pathways in which p38 MAPK, ERK 1/2, or NF-kB are involved [96]
Myricetin SubClass: Flavonols Class: Flavonoids Family: Polyphenols IC_50_: 1.2 µmol/L	Inhibition of DNMT activity in a concentration-dependent manner [20] Indirect modulation of deacetylation to induce *HIF-1α* expression and suppress *cMyc* and *β-catenin* expression [98]	Juice concentrate black currant (20.85), Fennel leaves (19.80), Goji berry dried (11.40), Arugula (7.92), Carob flour (6.73), Cranberries (6.63), Thistle (3.60), Ribes Nigrum (6.18), Been pollen (3.34), Swiss Chard (2.20), Red onion (2.16), Blueberries (1.30), Corn poppy (*Papaver rhoeas*) (1.10), Tea green (1.00), Blackberry (0.67), Black tea (0.45), Strawberry (0.35), Broccoli (0.06), Apple (0.01), Plums Black Diamond (0.01)	Prevention of cardiovascular disorders, anticancer ability, and reduction in blood lipid levels, blood pressure, and both diabetes and bacteriostasis complications [4]

HDAC: histone deacetylase; DNMT: DNA methyltransferase; HMT: histone methyltransferase; HAT: histone acetyltransferase; miRNA: microRNA; SIRT: sirtuin.

### 3.3. Flavones and Flavanones

Flavones, characterized by the backbone of 2-phenylchromen-4-one, are present in herbs like parsley, celery, thyme, oregano, and cereal species. Among the most common flavones are apigenin, luteolin, baicalein, and tangerine [100].

It has been reported that apigenin, contained in chamomile, parsley, and celery, modifies chromatin architecture, leading to inhibition of class I HDACs activity in human prostate tumor cells, causing cell cycle arrest and apoptosis, ultimately reducing cell growth (Table 3). Apigenin blocks the proliferation and growth of breast cancer cells through *p21* transcription associated with the acetylation of histone H3 [68,101]. Furthermore, apigenin, as well as luteolin, inhibits the activity of 5-cytosine DNMTs, including DNMT1, -3a, and -3b, in skin epidermal cells, along with the silencing of *nuclear factor erythroid 2-related factor 2* (*Nrf2*), inducing antioxidant and anticancer mechanisms [67]. Lastly, it induces the expression of both miR-16 and miR215-5p, exerting an anticancer role in glioma and colon cancer [7].

Luteolin, commonly found in peppers, peppermint, rosemary, carrots, and thyme, has several pharmacological properties, and it is used against diabetes and Alzheimer’s disorder (Table 3) [4]. It epigenetically regulates the expression of MMP9 and β-catenin, inhibiting proliferation and metastasis in breast tumors and inverting the epithelial-to-mesenchymal transition, respectively [102]. Luteolin also reduces the expression of DNMT1 and HDACs in a dose-dependent manner and downregulates calpain and UHRF1 in colorectal tumor cells, thus promoting apoptosis [69]. In prostate cancer cells, luteolin modifies the acetylation pattern of the gene promoter histone and inhibits the expression of 22 key genes involved in the cell cycle pathway [103]. Moreover, it regulates the expression of several miRNAs in different tumor cell lines [4].

Flavanones, unlike flavones, contain a saturated double bond between positions 2 and 3 (Figure 2). These compounds can be mainly found in the peel of citrus fruits and include a variety of phytochemicals, such as hesperetin, naringenin, naringin, and eriodictyol [104].

Hesperetin is a citrus flavanone that exerts its epigenetic regulation, diminishing the methylation of histone H3K79 to reduce metastasis recurrence (Table 3) [105].

Naringenin is a common citrus flavonoid and represents the aglycone form of naringin. Among their pharmacological properties, they have antidiabetic, liver- and heart-protective, antiviral, antioxidant, and antitumor effects. It is also used in sepsis treatment (Table 3) [106]. In neuroblastoma cancer cells, it promotes apoptosis, preserving the neighboring healthy cells due to its inhibition of HDAC activity. Moreover, both naringenin and hesperetin inhibit the activity of DNMTs [70]. Lastly, in human colon adenocarcinoma, naringenin can reduce the expression of several miRNAs, including miR-17-3p and miR-25-5p, involved in anti-inflammatory and antioxidant mechanisms, resulting in the upregulation of both glutathione peroxidase (GPX) 2 and manganese superoxide dismutase (SOD) [107].

**Table 3:** Epigenetic effects of flavones and flavanones, and their role in pathological conditions.

Compounds FLAVONES and FLAVANONES	Epigenetic Effects	High Content Food Quantity mg/100 g	Role in Pathological Conditions
Apigenin SubClass: Flavones Class: Flavonoids Family: Polyphenols Dosage: 500–1000 mg	Inhibition of class I HDAC activity in prostate tumor cells [20,68] Acetylation of H3 histone in breast tumor cells [101] Inhibition of the activity of 5-cytosine DNMTs and silencing of *NRF2* in skin epidermal cells [67] Induction of the expression of miR-16 and miR215-5p in glioma and colon cancer [7]	Parsley dried (4503.50), Celery seed (78.65), Vine Spinach (62.20), Kumquat (*Citrus japonica*) (21.87), Celery heart green (19.1), Red onion (0.24)	Inhibition of cell growth and promotion of cell cycle arrest and apoptosis in human prostate cancer cells [108] Blocking of the proliferation and growth of breast tumor cells through *p21* transcription [101] Antioxidant and anticancer effects [67]
Luteolin SubClass: Flavones Class: Flavonoids Family: Polyphenols	Inhibition of DNMT1 and HDAC enzymes in a dose-dependent manner in colorectal tumor cells [69] Modification of the acetylation pattern of the gene promoter histone in prostate cancer cells [103] Regulation of the expression of different miRNAs in several tumor cell lines [4]	Juniper berries ripe (*Juniperus communis* L.) (69.05), Parsley dried (19.75), Red onion (0.16)	Use against diabetes and Alzheimer’s disorder [4] Inhibition of proliferation and metastasis in breast tumors by regulating MMP9 expression and reversal of the epithelial-to-mesenchymal transition through the regulation of β-catenin expression [102] Promotion of apoptosis in colorectal tumor cells [69]
Hesperetin SubClass: Flavanones Class: Flavonoids Family: Polyphenols	Reduction in methylation of the histone H3K79 in gastric cancer cells [105] Inhibition of DNMT activity [70]	Grapefruit (1.50), Citrus paradise (1.50)	Anticancer effects by reducing metastasis recurrence [20]
Naringenin SubClass: Flavanones Class: Flavonoids Family: Polyphenols Dose: 500–1000 mg/day	Inhibition of DNMT and HDAC activity [70] Reduction in the expression of several miRNAs involved in anti-inflammatory and antioxidant mechanisms (including miR-17-3p and miR-25-5p) in human colon adenocarcinoma to upregulate GPX and SOD expression [107])	Grapefruit (53.0), Citrus paradise (53.0)	Antidiabetic, liver and heart-protective, antiviral, antioxidant, and antitumor effects; use in sepsis treatment [106] Promotion of apoptosis in neuroblastoma cancer cells [70]

HDAC: histone deacetylase; DNMT: DNA methyltransferase; miRNA: microRNA.

### 3.4. Isoflavones and Anthocyanidins

Isoflavones are characterized by a 3-phenylchromen-4-one skeleton in which the hydrogen at position 3 is replaced by phenyl (Figure 2). They are mainly contained in legumes, soy foods, soybeans, and fava beans. Genistein, daidzein, and glycitein are the main known isoflavonoids [109]. In particular, genistein and daidzein show a chemical and conformational structure similar to 17β-estradiol, thus allowing their interaction with estrogen receptors and their consequent phytoestrogenic property [110]. In addition to their phytoestrogenic role, isoflavones can also be employed against cardiovascular disorders, tumors, or other diseases associated with hormone dysfunction [4]. Indeed, several studies conducted in Asian countries have demonstrated that isoflavone consumption diminishes breast tumor risk, whereas no association has been detected within Western countries [111,112]. Considering that isoflavone intake is greater in Japan, Korea, and China compared to other countries, this discrepancy could be related to the ingested dose, which represents a pivotal factor able to influence their effect on tumor prevention and mortality [113].

Genistein, mainly present in soy protein and soybeans, is the flavonoid with the strongest antitumor and anti-proliferative effect against several tumors both in vitro and in vivo, thanks to its chemopreventive properties (Table 4) [114]. In prostate cell lines, genistein reduces DNA methylation at different gene promoters (such as *GSTP1* and *BRCA1*), along with a genome-wide modulation of DNA methylation patterns [115]. It promotes the inactivation of DNMT1, DNMT3a, and DNMT3b, leading to the increased expression of several tumor suppressor genes [116]. Its potential is not only related to DNMT enzyme inactivation but also to post-translational histone modifications, including HAT activation, histone demethylation, and SIRT inhibition [20]. In breast cancer, genistein enhances the ERα expression, and its combination with trichostatin A, a known HDACi, further increases ERα expression by promoting histone acetylation and inhibiting DNMT1 expression [117]. In ER-negative breast tumors, genistein reduces *BRCA1* methylation, thus resulting in *BRCA1* activation, which is associated with aryl hydrocarbon receptor (AhR) action inhibition [118]. Furthermore, it modulates the expression of several miRNAs in different cancers, including the activation of the miR-34a/RTCB axis in both head and neck tumors, which results in reactive oxygen species (ROS)-mediated apoptosis and reduction in epithelial-mesenchymal transition, counteracting tumor growth and cell cancer proliferation [119].

Daidzein, like genistein, can inhibit DNMT enzymes and activate HAT activity (Table 4) [67]. Specifically, daidzein inhibits the methylation of tumor suppressor gene promoters and genes involved in p53 and NF-kB pathways within human prostate tumor cells [4]. In breast tumors, combined with genistein, daidzein decreases the methylation of both *BRCA1* and *BRCA2* promoters, thus enhancing the BRCA1 and BRCA2 protein levels, and inhibits the MeCP2 expression associated with the entire genome methylation status [118].

Anthocyanidins, based on the flavylium cation, are water-soluble molecules responsible for the red and blue colors of plants. They are commonly present in grapes, cherries, and berries. They are flavonoid aglycones, and their glycosides are known as anthocyanins. Anthocyanidins exert beneficial effects, like anticancer, antioxidative, and vision-protective functions, and they are also used in the prevention and treatment of both type-2 diabetes and obesity. Delphinidin and cyanidin are the most common anthocyanidins contained in vegetables and fruits like blueberry, but there are also pelargonidin and malvidin [4,120].

Delphinidin is responsible for epigenetic regulation in different cancers, activating the Nrf2-ARE pathway due to the demethylation of several CpG sites within the *Nrf2* promoter (Table 4) [76]. In prostate tumor cells, it induces an enhancement in caspase-3, -7, and -8 expression, together with a boosted HDAC (mainly class I HDACs) activity. Moreover, delphinidin upregulates different pro-apoptotic genes, whereas downregulates anti-apoptotic ones [75].

**Table 4:** Epigenetic effects of isoflavones and anthocyanidins, and their role in pathological conditions.

Compounds ISOFLAVONES and ANTHOCYANIDINS	Epigenetic Effects	High Content Food Quantity mg/100 g	Role in Pathological Conditions
Genistein SubClass: Isoflavones Class: Flavonoids Family: Polyphenols (phytoestrogen group)	Activation of HAT activity and histone demethylation [73,74] Inhibition of DNMT enzymes [72] Modulation of several miRNAs in cancer [119]	Soy flour (89.42), Instant beverage Soy powder (62.18), Soy protein drink (42.91), Soy milk (42.85), Soybean (39.57), Natto (37.66), Tempeh (36.15), Miso (23.24), Green soybeans (22.57), Soy fiber (21.68) Soybean mature seeds (18.77), Tofu (16.01) Soy Yogurt (16.59)	Antitumor and antiproliferative effects [114] Suppressor of oncogene antiestrogenic activity [121] Induction of ROS-mediated apoptosis and reduction in epithelial-mesenchymal transition in both head and neck tumors, thus inhibiting tumor growth and proliferation [119]
Daidzein SubClass: Isoflavones Class: Flavonoids Family: Polyphenol (phytoestrogen group)	Activation of HAT activity [122] Inhibition of DNMT enzymes [115]	Soy flour (67.69), Soy Milk, dried (40.85), Instant beverage soy powder (40.07), Natto (33.22), Soy protein drink (27.98), Tempeh (22.66), Soybeans (21.75), Green soybeans (20.34), Soy fiber (18.80), Miso (16.43), Tofu (15.59), Soy Yogurt (13.77), Soybean mature seeds (12.86)	Antiestrogenic activity [123]
Delphinidin Subclass: Anthocyanidins Class: Flavonoids Family: Polyphenols	Inhibition of DNMT enzymes, which causes the activation of the Nrf2-ARE pathway [76] Increase in HDAC (in particular class I HDACs) activity [75]	Bilberry (97.59), Black currants (89.62), Eggplant (85.69), Blueberries (35.43), Black beans (18.50), Jambul (17.73), Red currants (9.32), Cranberries (7.67), Bananas (7.39), Pecans nuts (7.28), Jostaberry (6.61), Red onion (4.28), Red grapes (2.27)	Anticancer, antioxidative, and vision-protective functions; use in the prevention and treatment of type-2 diabetes and obesity [120] Upregulation of different pro-apoptotic genes and downregulation of anti-apoptotic ones [75]

HAT: histone acetyltransferase; DNMT: DNA methyltransferase; miRNA: microRNA; ROS: oxygen reactive species; HDAC: histone deacetylase.

### 3.5. Other Compounds

In addition to flavonoids, there are several other phytochemicals present in fruits, vegetables, spices, and beverages, able to exert epigenetic modifications, thus resulting in different effects, like anti-inflammatory, anticancer, and/or anti-microbial impact. Along with flavonoids, these phytochemicals might have the potential to be used as epigenetic drug targets against cancer and other pathological diseases, thanks to the reversibility of the induced epigenetic alterations [20,124].

Among natural compounds, curcumin, found in the plants of the *Curcumae longae* species, is a diferuloylmethane responsible for several beneficial effects on human health, like anticancer and anti-inflammatory properties. It is able to regulate a wide range of signaling pathways associated with proliferation, migration, apoptosis, inflammation, and metastasis (Table 5) [125]. Epigenetically, curcumin inhibits the activity of DNMT1 but also regulates both HAT and HDAC activities. Indeed, according to the different types of tumors, curcumin exerts diversified epigenetic effects [126]. It has been demonstrated to inhibit the HAT activity of p300/CBP, blocking the acetylation of both histones and non-histone proteins, including p53. Moreover, curcumin downregulates the activity of NF-kB and Notch1 in Raji cells through the inhibition of p300/CBP, HDAC1, and HDAC3, subsequently repressing cell proliferation [124]. Lastly, anticancer properties associated with curcumin are also related to its capacity to regulate both miRNA and lncRNA expression in tumor cells. Human pancreatic carcinoma cell lines treated with curcumin showed a changed expression of 29 miRNAs [127]. Instead, curcumin downregulates Bcl-2 expression through the induction of miR-15a and miR-16 in breast cancer, it enhances the expression of miR-34a in gastric cancer [128], and it inhibits the expression of miR-21 directly associated with tumor cell invasion and metastasis in human colon cancer cells [129]. However, curcumin is characterized by low bioavailability and poor pharmacokinetics. Therefore, the study and development of innovative curcumin analogs, such as refined preparations, to achieve better bioavailability and pharmacokinetics are still ongoing [130].

Folic acid, contained in beans, green vegetables, grains, cereals, and pasta, is included in the folate family and belongs to the methyl-metabolism pathway (Table 5). In contrast to naturally occurring folate (vitamin B9), folic acid is a synthetic metabolite that is ingested as a dietary supplement or in food. In order to obtain the biologically active 5-methyltetrahydrofolate, folic acid needs to be reduced to dihydrofolate through the action of dihydrofolate reductase enzyme within the liver and subsequently to tetrahydrofolate [131]. Hepatic DNA methylation status can be modified by a deficit of folic acid, which might be related to liver (but also breast, lung, brain, and colorectal) cancer [124].

Indole-3-carbinol (I3C) is a product of glucosinolate that is commonly contained in vegetables like cauliflower, cabbage, broccoli, and radish (Table 5). I3C can also be transformed into diindolylmethane (DIM). I3C and DIM can regulate different nuclear receptor-mediated signaling and kinases, thus promoting apoptotic mechanisms in several tumor cell lines [132]. Furthermore, DIM provokes proteasomal degradation of class I HDAC enzymes, leading to regulation of p21 and p27 expression and consequent inhibition of tumor growth [133]. DIM also regulates the expression of miRNAs, including miR-let-7e, miR-let-7b, miR-200b, and miR-200c [124].

Among the isothiocyanates, phenethyl isothiocyanate (PEITC) is found in cruciferous vegetables, and its main feature is its chemopreventive properties (Table 5). Indeed, PEITC can inhibit the proliferation and growth of different tumors and promote apoptotic mechanisms in several cancer cells [134]. In human prostate tumor cells, it has been demonstrated that PEITC inhibits HDAC activity through the enhanced de-methylation of the Ras-association domain family 1 isoform A (RASSF1A), whereas it induces a targeted histone acetylation and methylation [135]. Instead, in breast cancer cells, PEITC can target breast cancer stem cells through the epigenetic reactivation of *cadherin 1*, a tumor suppressor gene, by inhibiting DNMT and HDAC activity, thus demethylating its promoter site [136]. Moreover, PEITC regulates the expression of several miRNAs, like miR-125b, miR-26a, miR-192, miR-99, and miR-123, thus affecting NF-kB, Ras and TGF-β activation, and cell growth and apoptotic mechanisms [137].

Lycopene belongs to the tetra-terpenoid class and is commonly contained in ripe tomatoes, tomato products, and watermelons (Table 5). Indeed, lycopene is the pigment mainly responsible for their deep-red color. Its main ability is to reduce oxidative stress conditions thanks to its antioxidant properties. In human prostate, lung, liver, and breast cancer, lycopene inhibits tumor proliferation and growth through multiple molecular pathways [138,139]. Moreover, it protects against ultraviolet-related tumorigenesis by inhibiting inflammatory mechanisms and avoiding DNA structure injury [140]. Lastly, lycopene shows a demethylating impact on several promoters [124].

Resveratrol is naturally present in different plants, such as mulberries, peanuts, blueberries, and grapes, as well as in red wine (Table 5). It has anti-inflammatory and anticancer properties, influencing tumor cell proliferation, growth, invasion, and apoptosis, along with anti-aging benefits by modulating oxidative stress mechanisms. Its antioxidant properties are strictly related to free hydroxyl groups, which can donate hydrogen atoms to prevent cellular lipid peroxidation, in addition to resveratrol’s ability to promote antioxidative enzymes like catalase (CAT) and SOD [141]. It has been demonstrated that approximately 20 proteins, including Nrf2 and SIRT1, SIRT2 and SIRT3, could interact with resveratrol. Among different polyphenols, resveratrol is considered the strongest inducer of HDAC activity, thus influencing *SIRT* gene expression. Thanks to SIRT activation, resveratrol can positively regulate *AMPK*, which plays a crucial role in both aging mechanisms and energy metabolism and inhibits *NF-kB* expression, resulting in both anticancer and anti-inflammatory effects [142]. It has been reported that resveratrol promotes apoptotic mechanisms in colon, breast, and prostate tumor cell lines, as well as leukemia. In human breast tumors, this phytochemical enhances the expression of the *BRCA1* gene through H3 acetylation and AhR signal regulation, and in prostate cancer, it has been previously reported that resveratrol promotes tumor cell apoptosis by the deacetylation of *FOXO*. In breast cancer cells, resveratrol also increases the *ATP2A3* expression through the promotion of the H3 lysine 27 acetylation into the *ATP2A3* promoter, whereas it reduces methyl-DNA binding protein expression, such as MBD2 and MeCP2 [143]. Furthermore, in colon cancer cells, a reduction in the expression of different oncogenic miRNAs has been detected, which are able to regulate the RNaseIII Dicer1 [124]. It has been shown that after treatment with resveratrol, 22 miRNAs, like the tumor suppressor miR-663, have been upregulated, whereas 26 miRNAs, including miR-21 and miR-25, have been downregulated. Hence, resveratrol boots the TGFβ pathway but inhibits the transcriptional activity of its effector proteins called SMADs [144].

Lastly, sulforaphane is contained in cruciferous vegetables like cabbage, kale, and broccoli (Table 5). It is considered a chemopreventive and chemotherapeutic agent thanks to its ability to reduce oxidative stress levels, inhibit cell proliferation, and promote cell apoptosis in several tumor cell lines, including cervical, pancreatic, liver, lung, and ovarian cancers [145]. It has been reported that sulforaphane inhibits HDAC activity and regulates histone methylation through the promotion of the HDM named RBP2. It also diminishes histone H1 phosphorylation by increasing protein phosphatase 1β and 2A [146,147]. In human prostate cancer cells, HDAC inhibition, after sulforaphane treatment, is followed by a rise of global histone acetylation and consequent binding on *p21* and *Bax* gene promoters, leading to apoptotic processes. Together with *p21* and *Bax*, *FOXO* transcription factors can mediate sulforaphane-associated apoptosis. Sulforaphane also protects cells from ultraviolet-associated tumorigenesis [124,148]. Moreover, sulforaphane reduces the activity of DNMTs, mainly DNMT1, -3a, and -3b, in cervical, prostate, and breast tumor cell lines, restoring the expression of several silenced genes, like *cyclin D2* and *PTEN*, by demethylating their promoters [149,150]. Sulforaphane enhances the expression of several cytoprotective genes, such as *CAT*, *glutathione S-transferase (GST)*, and *SOD*, through the epigenetic reactivation of the Nrf2 pathway, which is involved in oxidative stress response and anticancer mechanisms [147]. Combined with EGCG, sulforaphane reduces breast tumor cell proliferation through the inhibition of both DNMT1 and HDAC1 activity, which increases *ERα* expression compared to single treatments [151]. Therefore, sulforaphane and EGCG intake diminishes the onset of ER-negative breast tumors and stimulates cancer sensitivity to tamoxifen [152]. Furthermore, in breast cancer cells, its combination with genistein induces HDAC activity inhibition and reduces hTERT protein levels than a single treatment [153]. Lastly, different miRNAs, including miR-21, miR200c, and miR-616-5p, have been demonstrated to be regulated by sulforaphane in several human tumors [147].

**Table 5:** Epigenetic effects of other phytochemicals and their role in pathological conditions.

OTHER PHYTOCHEMICALS	Epigenetic Effects	High Content Food	Role in Pathological Conditions
Curcumin Dosage: Curcuma longa powered 500 mg/day. Dosage Indian cook: 1–2 g/day	Regulation of both HAT and HDAC activities [126] Inhibition of DNMT activity [154] Inhibition of oncogenic miRNAs and promotion of tumor suppressor miRNAs [127]	*Curcuma longa* powder contains 2% curcumin; 1 g Curcuma contains 20 mg curcumin; 1 g Curry contains 2.9 mg curcumin	Modulation of intracellular pathways implicated in inflammation, proliferation, invasion, survival, and apoptosis in cancer [125] Cancer prevention and suppressor [127,129] Toxic effects at high doses in normal cells like human dermal fibroblasts, resulting in a pronounced arrest of cell cycle progression and higher levels of cell death [155]
Folic Acid Family: Folate The recommended daily intake of folate for adults is about 400 µg.	A key element in the methyl-metabolism pathway [156] Regulation of the hepatic DNA methylation status [157]	Breakfast cereals Fortified (100–400 µg per 100 g), Peanuts (240 µg per 100 g), Black-eyed Peas (210 µg per 100 g), Fortified pasta (100–200 µg per 100 g), Spinach (194 µg per 100 g), Lentils (181 µg per 100 g), Chickpeas (*Cicer arietinum*) (172 µg per 100 g), Asparagus (149 µg per 100 g) Brussels Sprouts (140 µg per 100 g), Beetroot (*Beta vulgaris subsp. vulgaris*) (109 µg per 100 g), Lettuce (136 µg per 100 g), Broccoli (108 µg per 100 g), Almonds (50 µg per 100 g), Grains (20–40 µg per 100 g), Oranges (30 µg per 100 g), Eggs (22–47 µg per 100 g.), Bananas (20 µg per 100 g), Pasta (not fortified) (10–20 µg per 100 g)	Deficiency can modify hepatic DNA methylation patterns and induce liver (but also breast, lung, brain, and colorectal) cancer [158]. Antioxidant and pro-oxidant effects [159]
Indole-3-carbinol (I3C) and diindolylmethane (DIM)	Proteasomal degradation of class I HDAC enzymes [133] Modulation of the expression of several miRNAs in cancer [160]	*Cruciferae family* (*Brassicaceae*) as Broccoli (15–25 mg/100 g), Cabbage (10–15 mg/100 g), Cauliflower (20–40 mg/100 g), Brussels Sprouts (40–50 mg/100 g), Mustard (10–15 mg/100 g), radish (all of *Brassica genus*) (1–4 mg/100 g)	Attenuation of symptoms of cigarette smoke [161] Anticancer effects, including promotion of apoptotic mechanisms in several tumor cell lines [132]
Phenethyl isothiocyanate (PEITC) Family: Isothiocyanates	Inhibition of HDAC and DNMT activity [136] Induction of targeted histone acetylation and methylation in human prostate tumor cells [135] Regulation of the expression of several miRNAs in cancer [137]	*Cruciferae family* as Watercress, Cauliflower, Cabbage, Cress, Bok Choy (*Brassica rapa subsp. Chinensis*) (Chinese cabbage) (10–150 mg per 100 g); Radishes (*Raphanus sativus*) (1–4 mg per 100 g); Arugula (1–2 mg per 100 g).	Inhibition of carcinogenic processes and regulation of stress response and inflammation [134]
Lycopene Family: Tetraterpenoid	Inhibition of DNMTs [139]	Tomato (2.5–4.5 mg per 100 g) Watermelon (4–7 mg per 100 g) Pink Grapefruit (1–3 mg per 100 g), Pink Guava (5–6 mg per 100 g), Papaya (2–3 mg per 100 g)	Antioxidant capacity, DNA protection from oxidative damage, reduction in tumor growth and proliferation in breast, prostate, liver and lung cancer [138] Protective agent against ultraviolet-related tumorigenesis [140]
Resveratrol Family: Polyphenols	Promotion of HDAC activity [162] Resveratrol targets: SIRT1, -2 and -3 [162] H3 acetylation in breast cancer, enhancing the expression of BRCA1 [144] FOXO deacetylation in prostate cancer [163] Reduction in the expression of different oncogenic miRNAs in colon cancer cells [144]	Red grape skin (0.5–1.5 mg per 100 g), Red wine (0.2–5.8 mg per 100 mL), Blueberry, Mulberry, Cranberry (0.02–0.06 mg per 100 g), and Peanut (0.1–0.3 mg per 100 g)	Anti-inflammatory, anti-aging, and anticancer properties, which influence tumor cell proliferation, growth, invasion, and apoptosis [141] Promotion of apoptotic mechanisms in colon, breast, prostate tumor cells and leukemia [124] Anti-aging potential and suppression of mouse skin cancer in combination with tea polyphenols [162,164]
Sulforaphane Family: Isothiocyanate	Inhibition of HDAC activity, regulation of histone methylation, and reduction in H1 histone phosphorylation [146] Inhibition of the activity of DNMTs, mainly DNMT1, -3a, and -3b, in cervical, prostate, and breast tumor cell lines [149,150] Regulation of the expression of several miRNAs in different human tumors [137,147]	Broccoli Sprouts (around 30–100 mg per 100 g), *Cruciferae* as Cabbage, Brussels Sprouts, Broccoli, Kale (*Brassica oleracea var. Sabellica*) (0.1–2.5 mg per 100 g)	Reduction in oxidative stress levels, inhibition of cell proliferation, and induction of cell apoptosis in cervical, pancreatic, liver, lung, and ovarian cancers [145] Promotion of the expression of several cytoprotective genes (such as catalase, glutathione S-transferase, and superoxide dismutase) through the epigenetic reactivation of the Nrf2 pathway [147] Cell protection from ultraviolet-associated tumorigenesis [165]

HAT: histone acetyltransferase; HDAC: histone deacetylase; DNMT: DNA methyltransferase; miRNA: microRNA; SIRT: sirtuin.

## 4. Bioavailability of Bioactive Molecules

As reviewed, the biological properties of phytonutrients and their protective roles in molecular mechanisms of some human diseases and cancer have been demonstrated using different experimental models. The low bioavailability and bioaccessibility restrict the application and represent the major limitation [166]. The term bioavailability refers to the quantity of bioactive molecules that pass through the digestive tract, are absorbed, and reach the peripheral tissues in the intact or metabolized form to perform their bioactivity or to be stored. Bioaccessibility is defined as the proportion of a compound consumed in a meal that is released from the food matrix during digestion, in which the luminal content is accessible for absorption in the small intestine and transformed by the microbiota. Bioactivity represents the activity of the absorbed compounds or their metabolites at the cellular level, resulting in biological effects on the body. The evaluation of the bioavailability of polyphenols and other bioactive compounds has recently been gaining increasing interest in developing nutraceutical products. Molecular structure, water solubility, and composition of food matrix modulate bioaccessibility and digestibility. In addition, membrane transporters and metabolizing enzymes modulate the bioefficacy of phytonutrients. Even technological processes and/or cooking and heat treatments modulate bioavailability, and the mean plasma levels of several phytonutrients after absorption are low. It has also to be taken into account that individual responses could affect phytonutrient’s bioavailability and bioactivity. It is also well known that polyphenols and other bioactive molecules in the large intestine are biotransformed by gut microbiota. Recent advances have been made to improve the bioavailability of some molecules. As of today, new delivery strategies have been studied, including lipid carriers, nano-emulsions, molecular enhancers, and encapsulation systems [167,168]. Various delivery systems were developed for carotenoids [169] and polyphenols [170]. However, further studies are necessary. The health benefits of dietary phytonutrients must be demonstrated in humans at appropriate doses, and well-controlled trials must be planned.

## 5. Phytochemicals and Traditional Anticancer Therapies: A Comparative Analysis Including Clinical Studies

The use of phytochemicals to fight cancer represents an innovative and expanding strategy. The goal is to overcome some of the limitations of traditional therapies, such as the low accessibility to tumor burden, the high dosage requirement, and their non-selective action [171].

Conventional anticancer strategies include chemotherapy, radiotherapy, and immunotherapy, but epigenetic drugs (epidrugs) are also increasingly being used. In particular, epidrugs exploit the reversible nature of epigenetic modifications, thus resetting the tumor epigenome. To date, the United States FDA (USFDA) has approved both DNMTi and HDACi [172]. Among DNMTi, 5-azacitidine (Vidaza, Azacitidine) and 5-aza-2′-deoxycytidine (decitabine (DAC), Dacogen) have been approved for myelodysplastic syndromes and acute myeloid leukemia treatment by reactivating silenced cancer suppressor genes [173]. Besides DNMTi, HDACi also shows promising anticancer properties through the reactivation of silenced cancer suppressor genes, thus promoting apoptotic mechanisms, inhibiting cell cycle arrest and DNA repair, and interfering with angiogenesis. At present, four HDACi have been approved by the USFDA: Vorinostat (SAHA) for the cutaneous T cell lymphoma treatment, Belinostat against peripheral T cell lymphomas, Romidepsin for both these diseases and Panobinostat for drug-resistant multiple myeloma treatment [174,175]. Instead, the only HDACi that was clinically approved in Europe is Panobinostat. Furthermore, the efficacy of DNMTi and HDACi can be improved by combining them or using each with other therapies, especially for solid tumors, as demonstrated by several trials. Indeed, epidrugs may be useful in sensitizing to traditional chemotherapy or immunotherapy [176]. Several preclinical analyses have demonstrated the anticancer potential of HDACi as both monotherapy and combinatory strategies in different solid tumors [177]. However, the subsequent clinical studies display positive results, with some exceptions due to the imperfections associated with preclinical research and the limitations of epigenetic strategies [27]. Indeed, the action of epidrugs is not locus-specific and induces large-scale modifications in the epigenome, also activating the expression of oncogenes and different off-target and adverse effects. Moreover, they display low permeability and solubility, making the investigation of alternative and innovative approaches essential [178]. Hence, there is a critical need to investigate the current state of clinical studies concerning HDACi efficacy and safety better and discover new potential strategies.

Among emerging anticancer approaches, evidence of the efficacy of flavonoids and other functional foods as innovative and alternative compounds with DNMTi and HDACi activity is constantly growing. Indeed, they display activity against several modifications in the intricate epigenetic network, from noncoding RNA to histone modifications [4]. After promising in vitro and in vivo results of flavonoid’s impact on epigenetics, several clinical trials have been conducted on flavonoids used alone or combined with other drugs [171]. The clinical efficacy of catechin, EGCG, and genistein has been extensively evaluated in several solid cancers, including lung, colorectal, breast, and prostate tumors, with encouraging results. In particular, green tea catechin showed anticancer potential against prostate cancer (Phase I) [171], colon cancer (Phase II) [179], breast cancer (Phase II) [180], bladder cancer (Phase II) [181], lung cancer (Phase II) [182], cervical tumor (Phase II) [183] and skin cancer [184]. EGCG was found effective for breast (Phase II) [185], lung (Phase II) [186], and colorectal tumor (Phase I) [187] treatment. Also, genistein against colon and rectal (Phase I/II) [188], breast (Phase II) and prostate cancer (Phase II), leukemia and lymphoma (Phase I/II), and quercetin for prostate (Phase I), and squamous cell cancer (Phase II) treatment have been clinically tested. Some of these clinical trials are still ongoing [171,189]. Promising results have also been obtained using daidzein for prostate cancer treatment (Phase II) [189]. Moreover, in order to overcome the poor solubility, quick metabolism, and low absorption within the gastrointestinal system, the nano-formulation of flavonoids is being investigated. Nanocarriers of catechins, fisetin, quercetin, and apigenin showed promising in vitro and in vivo results, and one clinical trial using nano-formulated luteolin has been launched for the treatment of tongue neoplasm [171,190]. However, even though clinical analysis performed on flavonoids showed encouraging results, the existing evidence for their clinical employment is still insufficient. Furthermore, the clinical trials performed are restricted by several limitations, such as the low number of participants. Therefore, additional clinical studies may be needed to further confirm the potential of functional foods for cancer treatment and develop innovative approaches (including combined administration and nano-drug carrier) to overcome bioavailability concerns.

## 6. Conclusions

The existing literature concerning dietary phytochemicals has demonstrated that compounds contained in functional foods have multifaceted epigenetic properties by modulating several epigenetic modifiers, such as HMTs, HATs, HDACs, DNMTs, and ncRNAs. Within this review, different flavonoids were investigated along with their epigenetic regulation and therapeutic effects, mainly against cancer. In addition to flavonoids, other phytochemicals characterized by potent anti-inflammatory, antioxidative, and anticancer properties were described. Thanks to the reversibility of the induced epigenetic modifications, phytochemicals show enormous potential in cancer prevention and treatment. Evaluation of the safety and efficacy of several natural compounds in preclinical and clinical trials is currently ongoing; the next crucial step would be to identify the most suitable doses of these natural compounds with the aim of obtaining favorable effects on humans. However, their potential is partially hindered by some limitations, like poor bioavailability, low water solubility, insufficient therapeutic index, and side effects on the liver. To overcome these restrictions, it is necessary to develop innovative strategies, such as nanomaterial encapsulation technology. Furthermore, combining plant-derived compounds with traditional chemo and/or radiotherapy may lead to better results and/or reduction in adverse effects. It is mandatory to continue deepening the global patterns of epigenetic modifications by phytochemicals with the aim of identifying new targets and attractive agents in fighting cancer.

## Data Availability

No new data were created or analyzed in this study.
